# An approach to fluoroless radiofrequency atrial fibrillation ablation

**DOI:** 10.3389/fcvm.2025.1524426

**Published:** 2025-01-22

**Authors:** Thaïs Pittet, Etienne Delacrétaz, Stéphane Cook, Hari Vivekanantham

**Affiliations:** Department of Cardiology, University and Hospital of Fribourg, Fribourg, Switzerland

**Keywords:** atrial fibrillation, electro-anatomical mapping, fluoroless radiofrequency ablation, intracardiac echocardiography, pulmonary vein isolation

## Abstract

Atrial fibrillation is the most prevalent arrhythmia with a lifetime risk of nearly 30%. It can be associated with reduced quality of life and complications such as heart failure and stroke. Pulmonary vein isolation (PVI) is the most effective treatment for rhythm control. It has initially been performed with fluoroscopic catheter guiding. The advent of three-dimensional (3D) electro-anatomical mapping has significantly reduced the need for fluoroscopy. More recently, intracardiac echography (ICE) techniques have been used to eliminate the need for x-rays. Additional advantages include providing electrophysiology lab personnel with a lead-free working environment and avoiding radiation exposure for both patients and physicians. ICE may also enhance the safety of the procedure by enabling a safe trans-septal puncture and the early recognition of cardiac tamponade. In this article, we present our approach to fluoroless radiofrequency PVI using ICE and 3D electro-anatomical mapping.

## Introduction

Atrial fibrillation (AF) is the most prevalent cardiac rhythm disorder affecting 1% of the Swiss population, and imposes a high burden on the health care system (1). This condition can cause debilitating symptoms with a negative impact on quality of life and increases the risk of stroke up to a fivefold (2). Pulmonary vein isolation (PVI) is the most effective treatment to achieve rhythm control, with proven efficacy in reducing arrhythmia recurrence and hospitalization (3). According to the most recent european guidelines, PVI should be considered the first-line approach for rhythm control in paroxysmal AF and in case of failure or intolerance to anti-arrhythmic drugs (4). Traditionally, fluoroscopy is required for catheter guiding and trans-septal (TS) access, which is a key step in the procedure. However, the emergence of non-fluoroscopic ablation techniques offers an alternative to avoid radiation exposure for both patients and healthcare professionals (5). We describe our approach to perform a fluoroless radiofrequency (RF) PVI procedure using intracardiac echography (ICE) and three-dimensional (3D) electro-anatomical mapping (EAM).

## Material and equipment

Our approach is aided by the CARTO™ 3 (Biosense Webster Inc., California, USA) EAM system. Although not mandatory for the fluoroless approach *per se*, we routinely use specific modules such as CARTOSOUND® and CARTOSOUND™ FAM (Biosense Webster Inc., California, USA), as detailed later on. Three sheaths are used: a CARTO VIZIGO™ medium curve bi-directional steerable sheath (Biosense Webster Inc., California, USA), a Swartz™ SL0 63 cm transseptal braided introducer (Abbott Medical, Plymouth, USA) and a standard 14–15 cm 10 Fr introducer. A BRK™ XS 71 cm needle (Abbott Medical, Plymouth, USA) is used for TS puncture. A SOUNDSTAR® eco (Biosense Webster Inc., California, USA) ICE catheter allows using the above-mentioned CARTOSOUND® features. For mapping and ablation we use an OCTARAY™ (Biosense Webster Inc., California, USA) multipolar multi-spline catheter and a THERMOCOOL SMARTTOUCH® SF (Biosense Webster Inc., California, USA) irrigated catheter, respectively.

## Methods

### Patient preparation

Preoperative cardiac imaging is not routinely requested, but may be performed at the physician's discretion, depending on the thromboembolic risk and the anticipated complexity of the anatomy. The patient is brought to the electrophysiology (EP) lab in a fasting state with uninterrupted oral anticoagulation intake. We typically perform the procedure under general anesthesia (GA), although deep sedation is a reasonable alternative.

### Venous access and guiding sheaths

Femoral venous access is obtained under ultrasound guidance using Seldinger's technique, and three guidewires are advanced into the femoral veins. The three sheaths are positioned from the lateral to the medial aspect of the right groin as follows: CARTO VIZIGO™ steerable sheath, Swartz™ SL0, and 10 Fr introducer. Subsequently, the CARTO VIZIGO™ and SL0 sheaths are advanced halfway towards the inferior vena cava if no resistance was met when advancing the respective guidewires. At this point, full or half-dose heparin is administered at the operator's discretion.

### Imaging with ICE

While advancing the ICE catheter through the 10 Fr sheath, a flat echo-free space is maintained ahead of the transducer. Some degree of anterior or posterior tilt may be required depending on venous angulation, to reach the inferior vena cava and avoid the hepatic vein. Views obtained from the ICE catheter can assist in advancing the guidewires through the sheath if needed.

**Figure 1:**
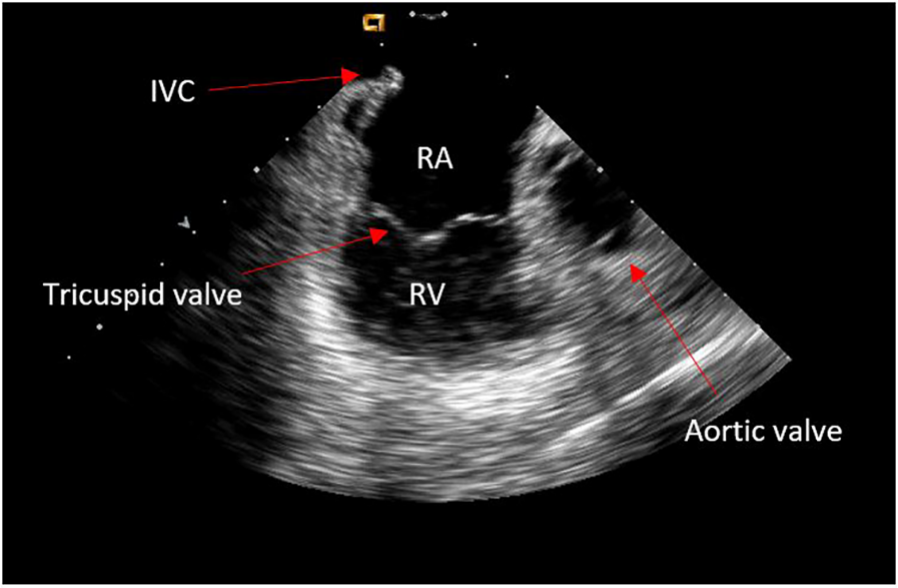
Home view.

**Figure 2:**
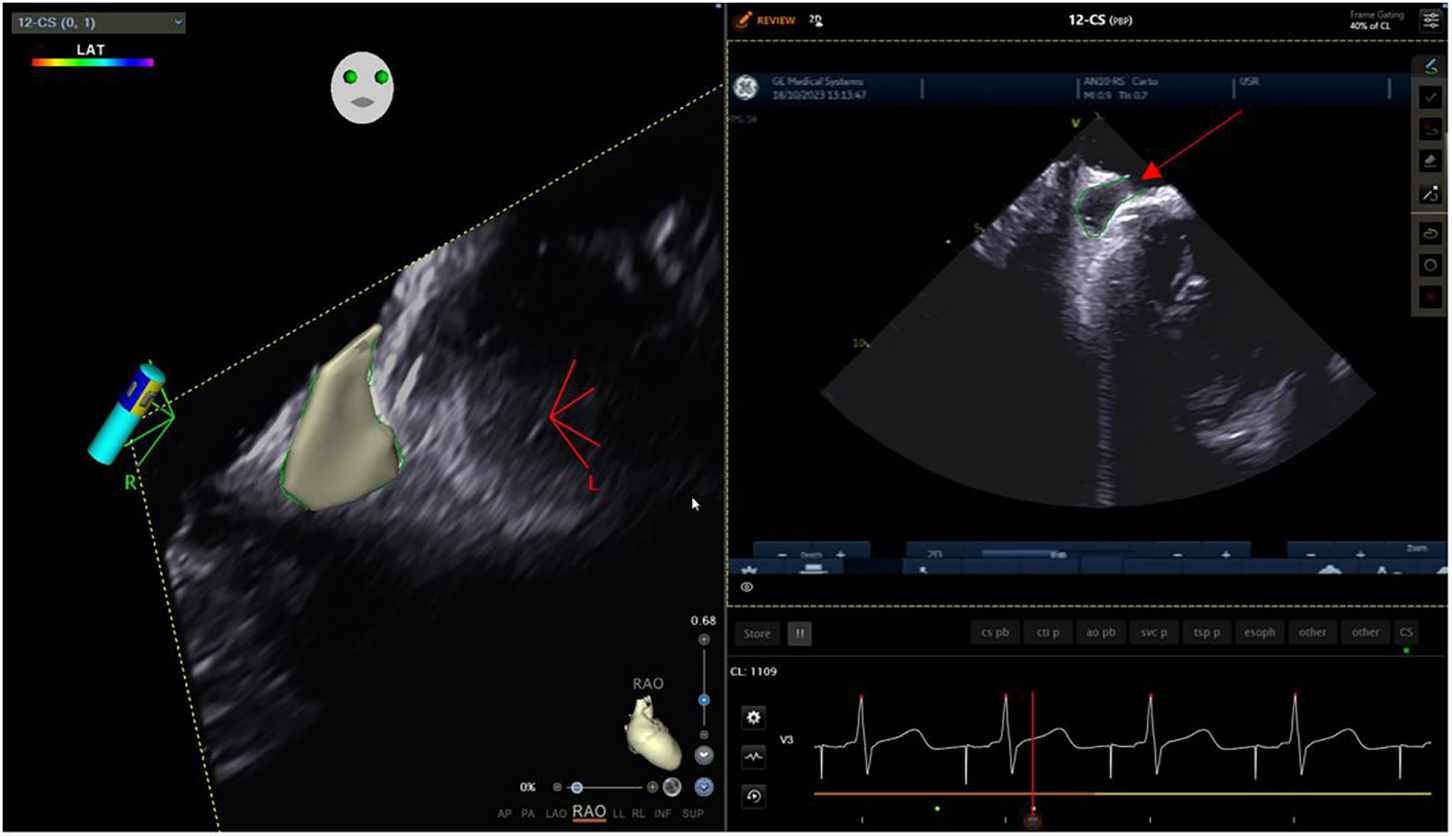
Coronary sinus ostium.

The “home view” from the RA shows the ICE probe facing the tricuspid valve and right ventricle (RV) without any anterior/posterior or right/left tilt (neutral position) (Figure 1). The presence or absence of pericardial effusion as well as aspect of the right heart cavities can be appreciated. From this point onward, the rotational wheel on the ICE handle is locked to maintain the desired deflection/tilt position. Following respiration gating, anatomy of key structures is collected using the CARTOSOUND® Module and manually tagged: the cavotricuspid isthmus (CTI) is visualized in the home view; a slight clockwise rotation will show the aortic root and valve; further clockwise rotation will reveal the coronary sinus (CS) ostium (Figure 2). The next steps will focus on visualization of the interatrial septum (IAS) and LA. From the home view, slight advancement and clockwise rotation past the aortic valve will bring both structures into view. The fossa ovalis (FO) can be carefully studied in preparation for the TS access, and the presence of an IAS aneurysm or a patent foramen ovale can be detected (color Doppler may also be used). Advancement and wedging of the ICE catheter on the IAS usually allows for the best visualization of LA structures.

### Reconstruction of the left atrium

**Figure 3:**
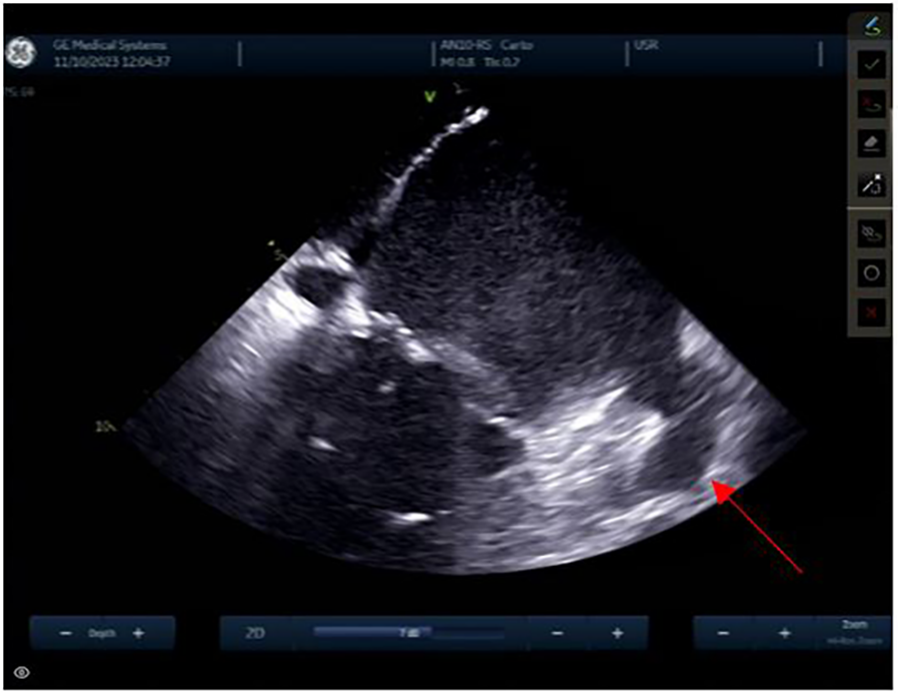
Left atrial appendage.

**Figure 4:**
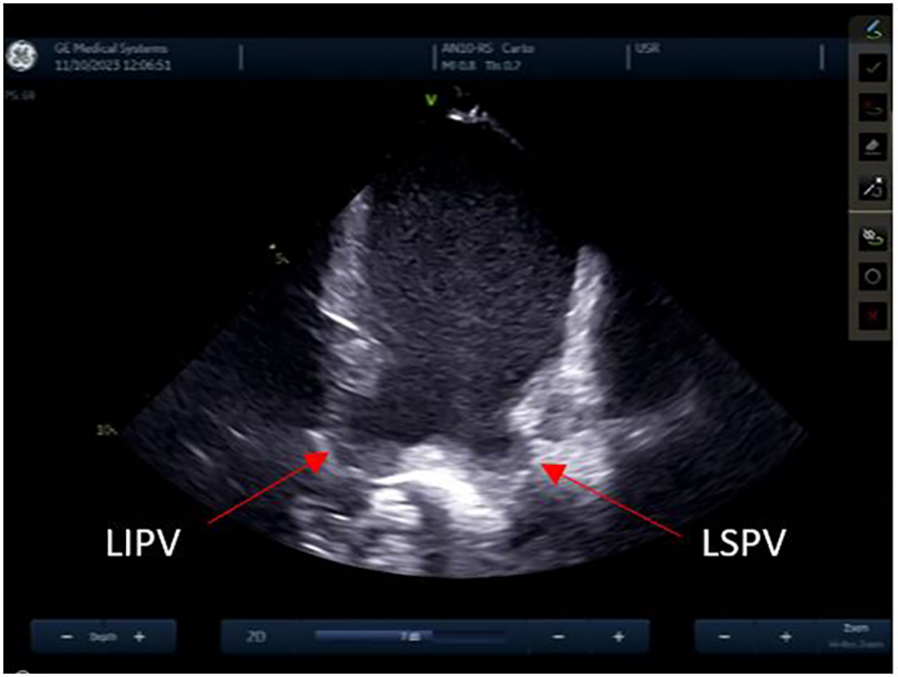
Left pulmonary veins.

**Figure 5:**
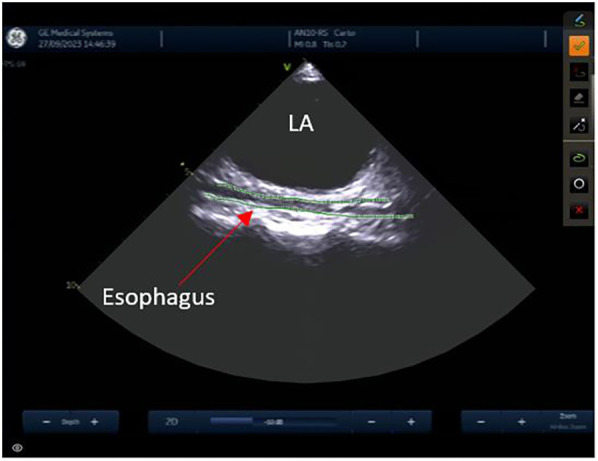
Esophagus.

The left atrial appendage (LAA), and the mitral valve annulus can be visualized with counterclockwise rotation (Figure 3). Clockwise rotation will sequentially reveal the left superior pulmonary vein (PV), left inferior PV (Figure 4), posterior wall of the LA, right inferior PV, and right superior PV. The esophagus may be seen behind the posterior LA wall and manually drawn with the CARTOSOUND® module (Figure 5). Advancement of a temperature probe in the esophagus can be guided by ICE, although we do not routinely use one. Finally, after returning to the home view, the ICE probe is advanced into the RV with anterior and slight counterclockwise tilt. A neutral position will then bring the probe's tip toward the outflow tract and reveal the left ventricle. Clockwise rotation and slight withdrawal of the probe, taking care not to fall back in the RA, will allow to assess the LAA for the presence of blood clot, and help define the ridge separating it from the left superior PV (Figure 3). The CARTOSOUND™ FAM module may be used to perform an automated LA anatomy reconstruction based on the ICE images. An ablation catheter is advanced into the heart through the CARTO VIZIGO™ sheath, under EAM guidance. Following catheter force calibration, a His signal is sought, guided by the anatomy collected through CARTOSOUND® (typically located at the aortic root level). Although this step is not crucial in the procedure, marking the central part of the heart may serve as an anatomical reference.

### Trans-septal puncture

**Figure 6:**
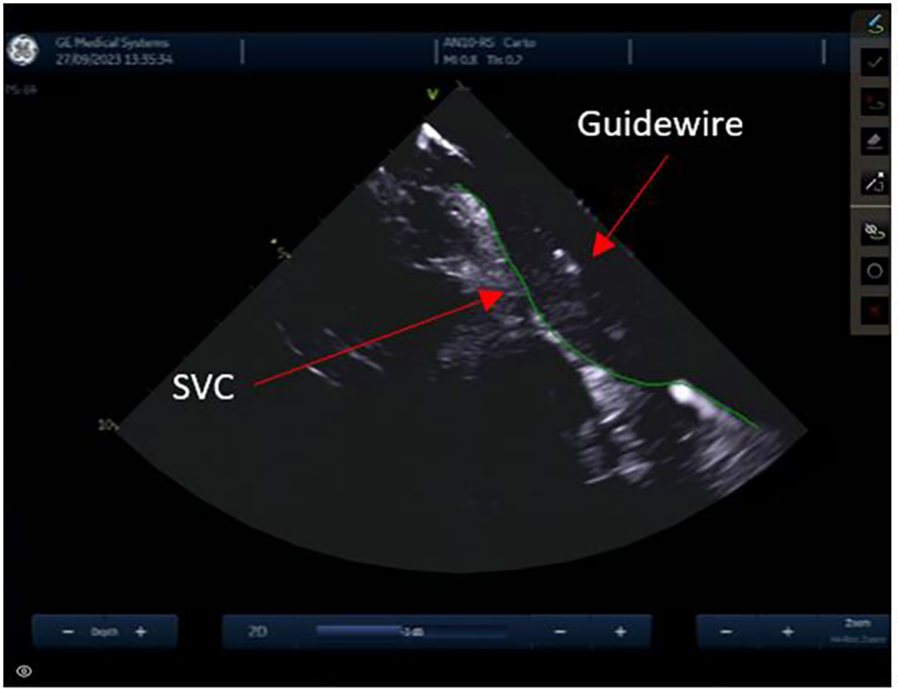
Superior vena cava.

**Figure 7:**
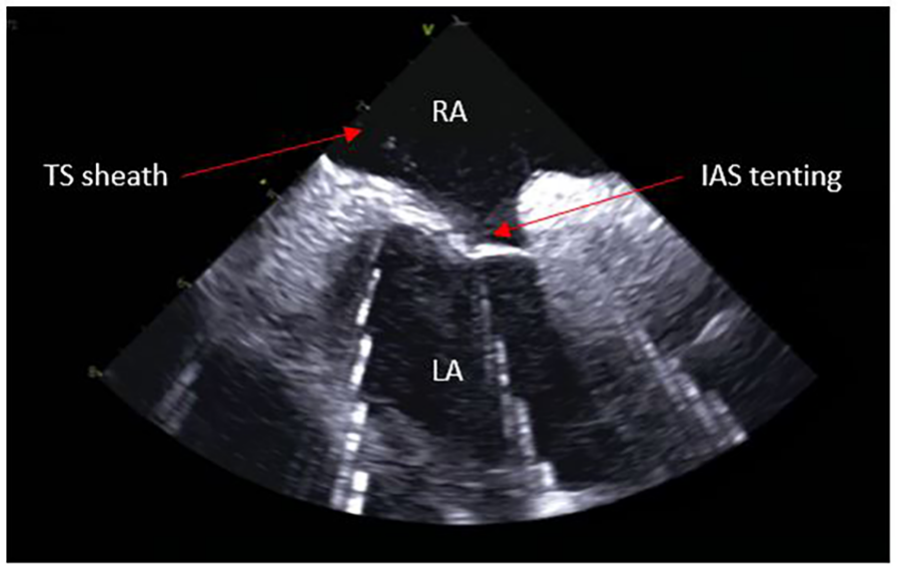
Trans-septal puncture.

From the IAS view, a posterior and right tilt will bring the superior vena cava (SVC) into view and allow for controlled advancement of the SL0 guidewire (Figure 6). The SL0 introducer will then be advanced into the SVC in a 3 to 4 o'clock angle of rotation. Following removal of the wire, saline injection can further confirm the sheath's placement by visualization of microbubbles. The TS needle is then advanced into the sheath with a 20 ml syringe filled with saline connected to its proximal port. The TS system (sheath, dilator, and needle) is gently withdrawn under ICE guidance. The ICE position may need to be adjusted to clearly see the drop into the IAS. This part may require a staff member to manipulate the ICE while the physician is handling the TS system with both hands. Once an appropriate TS position is obtained, with the tip of the guiding sheath facing the left PVs, the system is advanced to tent the FO, and the needle is extended outwards (Figure 7). A “pop” through the IAS can be felt and visualized. Injection of a small amount of saline through the needle confirms TS access with visualization of microbubbles in the LA. The dilator and sheath are slightly advanced to cover the needle, which is then withdrawn. The guidewire is advanced through the sheath and into one of the left PVs. Once the position of the wire is confirmed with ICE, the IAS is dilated with the sheath several times. The SL0 sheath is withdrawn in the RA, which will permit advancement of the ablation catheter to the LA and up the left PV, through the TS gap. The SL0 sheath can then be re-advanced to the LA to further dilate the IAS, before advancement of the CARTO VIZIGO™ sheath over the ablation catheter. The mapping catheter is finally introduced through the SL0. If only half heparin dose was given, the rest of it can now be administered.

### Electro-anatomical mapping and ablation

LA mapping greatly depends upon the operator's preference, available EAM system, and catheters used. As mentioned earlier, we perform the procedure with the support of the CARTO™ 3 EAM system and its specific modules. Mapping is performed in sinus rhythm and ablation is performed according to the CLOSE protocol (6). The mapping catheter is typically positioned in the superior PV while a wide area circumferential ablation is performed around the concomitant PV side. This allows for immediate visualization of PV disconnection. In case of bradycardia, pacing from the PV may be performed. As the procedure is performed under GA, we request high-frequency, low-tidal volume ventilation during ablation (7).

## Results

The use of ICE has several limitations. The SOUNDSTAR® ultrasound catheter requires a 10 Fr venous femoral access. This should not be considered as an additional venous access, as operators performing fluoroscopic guided TS commonly use a CS catheter (through femoral or jugular venous access) as a landmark, whereas it is not required based on our technique. In the event of CTI line ablation, we propose pacing from the LA floor to check for CTI block, as an alternative to proximal CS pacing.

Manipulating the ICE catheter is overall safe with very few mechanical complications. Debreceni et al. exposed similar complications rate between fluoroless and standard approach (8). In addition, Žižek et al. demonstrated the feasibility and safety of ICE and only raised awareness for patients with cardiac implantable electronic devices, as the trans-septal puncture can cause lead dislocation even with the use of ICE (9).

Mastering ICE, like any new technique, requires acquiring new skills and involves a learning curve. We suggest starting with a combination of fluoroscopy and ICE before transitioning to fluoroless PVI. Other limitations include catheterization issues because of venous tortuosity, and suboptimal visualization of intracardiac structures. Finally, the cost of the ICE catheter is significant. A cheaper alternative to the SOUNDSTAR® ICE catheter is the ACUSON AcuNav™ (Siemens Medical Solutions USA, Inc., Issaquah, USA). However, it doesn't offer the possibility to collect and reconstruct cardiac anatomy.

## Discussion

Radiation exposure in the medical field should be kept as low as reasonably achievable for patients and healthcare workers (10). Fluoroless trans-septal catheterization, LA mapping, and PVI is a widespread technique in North America which offers several advantages. ICE provides a safe method for performing the TS puncture by precisely visualising intracardiac structures, which is of utmost importance in case of complex anatomy. In addition, it allows for monitoring of potential complications throughout the procedure. As the standard ablation technique itself is already fluoroless thanks to the EAM, it has been established that the procedural success is similar with or without fluoroscopy (8). Transesophageal echocardiography (TEE) is an alternative to ICE but requests GA and a second physician. In addition, TEE exposes the patient to the risk of esophagial lesions. With the fluoroless approach, both the patient and the EP lab staff are protected from the cumulative and long-term harmful effects of radiations (11). Finally, it allows working in a lead-free environment. Indeed, the deleterious effects on the musculoskeletal system resulting from chronic lead apron use among cardiologists have been proven (12). We describe our standard work-flow for fluoroless RF PVI, although numerous alternatives exist. It is our preference to have two catheters in the LA to avoid catheter exchanges and to facilitate mapping during ablation if first-pass PVI is not achieved. When linear lesions are performed, validation of conduction block is facilitated. Differentiating near field from far field signals with mapping catheters during ablation (i.e., LAA) is also easier. With accumulating experience, each operator/center will develop the most suitable and well-adapted workflow.

## Data Availability

The original contributions presented in the study are included in the article, further inquiries can be directed to the corresponding author.
